# Long Exposure to a Diet Supplemented with Antioxidant and Anti-Inflammatory Probiotics Improves Sperm Quality and Progeny Survival in the Zebrafish Model

**DOI:** 10.3390/biom9080338

**Published:** 2019-08-03

**Authors:** David G. Valcarce, Marta F. Riesco, Juan M. Martínez-Vázquez, Vanesa Robles

**Affiliations:** 1 IEO, Spanish Institute of Oceanography, Planta de Cultivos El Bocal, Barrio Corbanera, 39012 Monte, Santander, Spain; 2 MODCELL Group, Department of Molecular Biology, Universidad de León, 24071 León, Spain

**Keywords:** spermatozoa, probiotics, embryo, reproduction, nutrition

## Abstract

The aim of the present experiment is to study the effects of oral ingestion of a mixture of two probiotic bacteria on sperm quality and progenies. Three homogeneous groups of juvenile zebrafish were created. Once having reached adulthood (3 months postfertilization; mpf), each group received different feeding regimens: a standard diet (control), a maltodextrin-supplemented diet (vehicle control), or a probiotic-supplemented diet (a mixture (1:1) of *Lactobacillus rhamnosus* CECT8361 and *Bifidobacterium longum* CECT7347). The feeding regime lasted 4.5 months. Growth parameters (weight and length) were determined at 3, 5, and 7.5 mpf. Sperm motility was evaluated using computer-assisted sperm analysis at 5 and 7.5 mpf. Progeny survival, hatching rate, and malformation rate were also evaluated. Results showed that probiotic-supplemented diet improved growth parameters compared with the standard diet. The highest percentage of motile spermatozoa was reported in the probiotic-fed group. Concomitantly, the percentage of fast sperm subpopulation was significantly lower in samples derived from control males. Furthermore, there was a significant difference in progeny survival between the probiotic-fed group and the control group at three developmental times (24 hours postfertilization (hpf), 5 days postfertilization (dpf) and 7 dpf). In conclusion, in zebrafish, prolonged ingestion of a mixture of *Lactobacillus rhamnosus* CECT8361 and *Bifidobacterium longum* CECT7347 has positive effects on growth, sperm quality, and progeny survival.

## 1. Introduction

Over the last three decades, the zebrafish (*Danio rerio*) has received increasing interest as a model system for studying vast fields of science due to its numerous advantages and characteristics. This small vertebrate presents cheap and easy maintenance and a high degree of similarity with the human genome [1]. Nowadays, zebrafish is used world-wide in evolutionary science [2], genetics [3], development [4], neuroscience [5], drug discovery [6], environmental monitoring [7], or human disease research [8,9]. This teleost presents useful developmental and physiological advantages for reproductive biology [10,11,12]. The short reproductive cycle, the optical transparency of the animal in the early developmental stages, and rapid development allow versatile approaches to be performed [13]. Reproductive functions and regulations between *D. rerio* and mammals are similar [14,15] and this fact has promoted it as a promising model organism in infertility and reproductive studies [10,16].

One potential approach to improve reproductive performance is diet. The adverse consequences of unbalanced diets on reproduction have been vastly reported [17,18,19] as well as positive correlations between optimized diets and reproductive performance [20]. Dietary supplements are noninvasive, cheap, and clinically applicable. The improvement of dietary factors can influence fertility and progeny development. Among the myriad of existing diet supplements, during the last decade, probiotics have swum into a well-accepted product by the consumers and animal producers. Probiotics have been defined as “live microorganisms that, when administered in adequate amounts, confer a health benefit on the host” [21] and may play a role as a positive oral supplement in the reproductive field.

This study investigated for the first time the effects of a long exposure (since the beginning of adulthood) to an oral ingestion of a mixture of two probiotic strains on sperm quality and offspring success and development. To do this, we used zebrafish, which, thanks to its rapid growth, short reproductive cycle, and ease in monitoring embryonic development, allowed us to carry out an experimental design prolonged over time. To validate our hypothesis, we used a combination of two strains—*Lactobacillus rhamnosus* CECT8361 and *Bifidobacterium longum* CECT7347—which have been proven to present antioxidant [22] and anti-inflammatory [23] properties.

The main goal of this study is to provide knowledge about new tools that may be applied prior to reproductive technologies, improving gamete quality and producing healthier offspring.

## 2. Materials and Methods 

### 2.1. Ethics Statement

The current study was performed according to the guidelines of the European Union Council (86/609/EU, modified by 2010/62/EU) following Spanish regulations (RD/1201/2005, abrogated by RD/2013) for the use of laboratory animals. All experimental protocols and procedures were approved (permission code: PI-10-16) by the institutional Animal Care and Use Committee at the Marine Culture Plant El Bocal of the Spanish Institute of Oceanography in Santander (Spain).

### 2.2. Model Organisms

Wild-type zebrafish (Ab strain) were raised and kept under standard laboratory conditions [24] in the Marine Culture Plant El Bocal zebrafish platform of the Spanish Institute of Oceanography in Santander (Spain). Fish used for the current experiment were maintained in a recirculation culture system (Aquatic Habitats, Apopka, FL, USA) maintained at 28 ± 1 °C on a 14 h light/10 h dark cycle. In any case, animals were anesthetized using a 110 mg/L 3-aminobenzoic acid ethyl ester (MS-222) solution. If animals reached a moribund condition, humane endpoint was applied with a lethal dose of anesthetic.

### 2.3. Study Design, Experimental Diets, and Feeding Regimes

**Figure 1:**
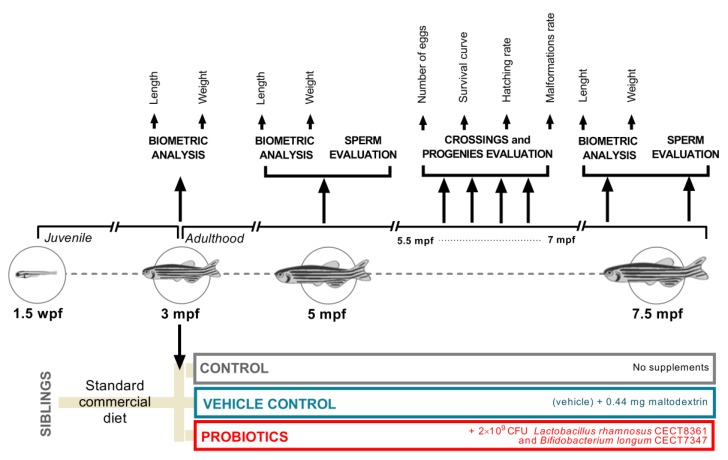
Design summary. wpf: weeks postfertilization; mpf: months postfertilization.

Nine adult spawning zebrafish from the same system tank with no visible signs of injuries, infections, or abnormal behavior were standard-crossed (1:2 ratio; male:female). Resulting progenies were checked for normal development, grouped in a single batch, and cultured following standard protocols. At 1.5 months after fertilization (mpf), the batch was randomly split into three homogeneous groups (initial population for each experimental group *n* = 30) (Figure 1). At 3 mpf (beginning of the adulthood in zebrafish), each group started to receive a different feeding regime from this time onwards: 1) the control group “CONTROL” ingested only commercial extruded feed optimized for adult zebrafish (Aquatic Animals; Apoka, FL, USA) consisting of 55% minimum crude protein, 15% min crude fat, 1.5% maximum crude fiber, and 12% maximum moisture; 2) the vehicle control group “MALTO” received two doses of 0.22 g of maltodextrin as a supplement to the commercial diet, and 3) the experimental group “PROBIO” ingested the commercial diet and a probiotic treatment consisting of a daily 2 × 10^9^ colony-forming unit (CFU) mixture (1:1) of lyophilized *Lactobacillus rhamnosus* CECT8361 and *Bifidobacterium longum* CECT7347 strains vehicled in 0.44 g of maltodextrin. In addition, to avoid any possible external contaminations, during the experiment, the experimental groups never received *Artemia* sp., a common practice in zebrafish larvae culture. Biopolis S.L. (Valencia, Spain) kindly provided the probiotic strains. The animals were fed twice a day, seven days/week, at 08:30 and 13:30 h, with the commercial extruded feed. To confirm the ingestion, supplements (vehicle or probiotic mixture) were provided to animals in rearing water 0.5 h before providing commercial diet. Feeding regimes were maintained until the end of the experiment.

### 2.4. Biometric Analysis

The growth trial lasted for 4.5 months. Before sampling, fish were fasted for at least 18 h. Animals were anesthetized individually, and when gill movements were clearly reduced (around 30 s), they were weighed using a microbalance (Mettler MT5, Mettler Toledo, Spain) to an accuracy of mg. Fish length (accuracy of 0.01 mm) was monitored using Adobe Photoshop CC 2018 software (using a conversion between pixel size and units of length). Pictures of each animal were taken under a stereomicroscope (Nikon SMZ-U; Nikon, Japan) with a scale reference and afterwards digitally analyzed. Biometric analyses were performed at time: 3, 5, and 7.5 mpf.

### 2.5. Collection of Germplasm

Anesthetized males were gently placed on a sponge, the urogenital pore was toweled, and gametes were expelled by gently stroking both sides of the fish abdomen with smooth flat glass forceps according to routine protocols. Collected ejaculates were diluted in 10 µL of buffered Hank’s solution (0.137 M NaCl; 5.4 mM KCl; 0.25 mM Na_2_HPO_4_; 0.44 mM KH_2_PO_4_; 1.3 mM CaCl_2_; 1.0 mM MgSO_4_; 4.2 mM NaHCO_3_) and stored in 1.5 mL centrifuge tubes at room temperature (RT), until analysis.

### 2.6. Progenies

Mating was performed with a sex ratio 1:2 (male:female) (siblings). Sex identification was assessed based on the sexual dimorphism and pigmentation. Reproduction performance trials began when fish were prone for sure mating intercourse (5.5 months-old). Fertilized eggs were recovered from breeding tanks after natural spawning on the onset of light in the zebrafish facility. Fertilized embryos were rinsed and then randomly distributed in Petri dishes (100–150 embryos/dish). The offspring resulting from each crossing were considered biological replicates. The total number of fertilized embryos was counted for each replicate. F1 animals were incubated at 28 ± 1 °C from 0 hours postfertilization (hpf) until 8 days postfertilization (dpf).

### 2.7. Progeny Evaluation

Dead embryos or larvae were removed from the dishes at 12 hpf and 1 to 7 dpf. The survival rate (percentage of live animals per initial batch) was evaluated daily until 7 dpf. Embryo and larvae development was scored under stereomicroscope (Nikon SMZ-U; Nikon, Japan). Hatching rate was established as the percentage of hatched larvae per live animals within the batch at 48 hpf and 72 hpf. Malformation rate was evaluated at 5 dpf (when morpohogenesis was fully completed [25]). Malformation rate was calculated as the percentage of noncanonical larvae per live animals within the batch at 5 dpf. 

### 2.8. Sperm Analysis

The sperm motility kinetics and concentration assessment were performed using computer-assisted sperm analysis (CASA). Diluted semen (1 μL) was dropped onto a Makler counting chamber (10 µm depth; Sefi Medical Instruments, Haifa, Israel). Thereafter, spermatozoa were activated with 9 µL of system water at 28 °C and analyzed with ISAS software (ISAS, Proiser R+D, S.L., Valencia, Spain). The CASA system consisted of an optical phase contrast Nikon Eclipse Ts2R microscope (Nikon, Tokyo, Japan) using a 10× objective equipped with Basler A312fc digital camera (Basler Vision Technologies, Ahrensburg, Germany) specifically set for fish spermatozoa (1 µm^2^ < particle area < 20 µm^2^; sperm characterization following curvilinear velocity (VCL): 10 µm/s < slow < 45 µm/s < medium < 100 µm/s < fast). To avoid skewing results derived from drifting and to corroborate an exact equal time of evaluation among samples, fields were captured 15 s after activation. Three fields per sample were evaluated. The sperm quality parameters included in our study were: 1) concentration; 2) motile spermatozoa (MOT,%); 3) progressive spermatozoa (P-MOT,%), considered spermatozoa which swim forward in 80% of a straight line; 4) curvilinear velocity (VCL, µm/s) defined as the time per average velocity of a sperm head along its actual curvilinear trajectory; and 5) straight-line velocity (VSL, µm/s) defined as the time per average velocity of a sperm head along the straight line between its first-detected position and its last position.

### 2.9. Statistical Analysis

Statistical analysis was carried out using Prism 8 (GraphPad Software, San Diego, CA, USA) and SPSS V. 22 (SPSS Inc, Chicago, IL, USA). Significance differences were considered with *p*-values < 0.0500. Data were submitted to Kolmogorov–Smirnov and Levene’s tests to verify normality and homogeneity of variances, respectively. Data were analyzed by one-way ANOVA (analysis of variance) or Kruskal–Wallis in non-normally distributed data. Results are expressed as the mean ± standard error of the mean (s.e.m). A principal component analysis was performed for the set of observed kinetic variables rendered by CASA. Embryo survival curves comparison was performed using Kaplan Meier functions. Significance of differences among survival curves was determined with the log rank test.

## 3. Results

### 3.1. Effects of Probiotic Ingestion on Biometric Parameters

**Figure 2:**
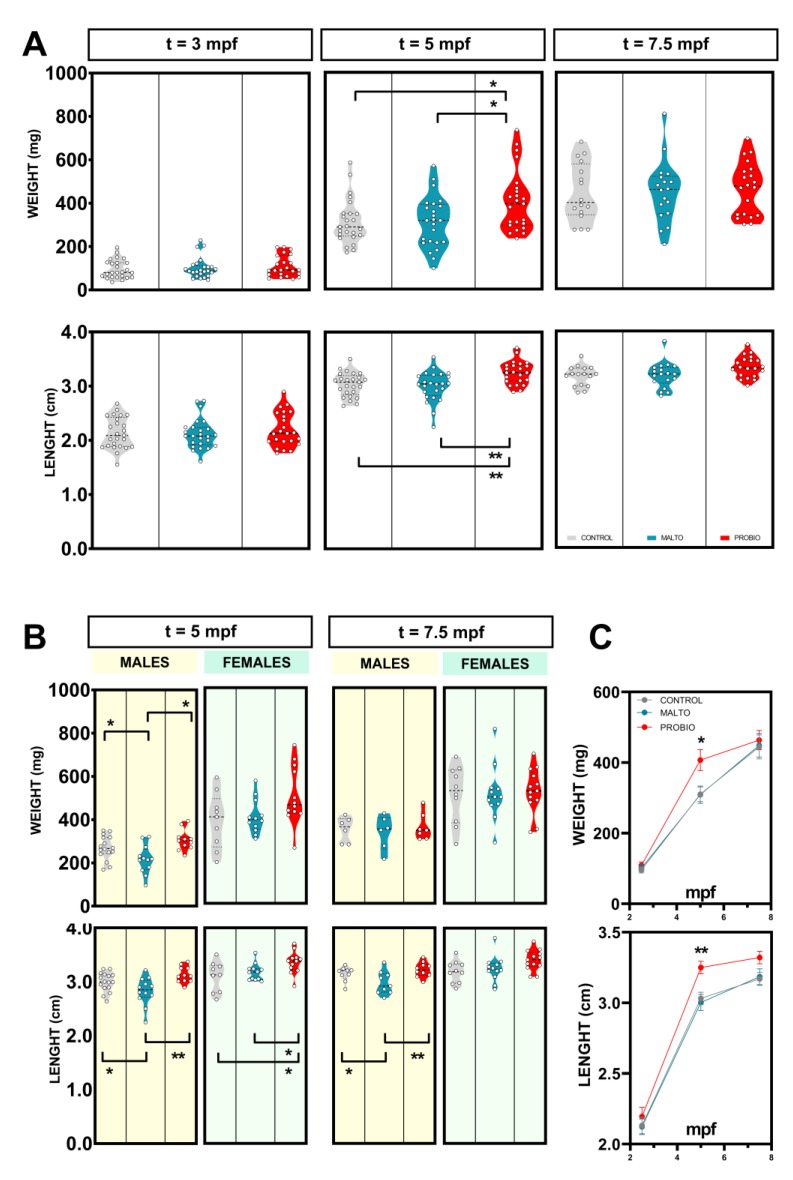
Feeding with probiotics promotes growth in zebrafish model. (**A**) Violin plots showing growth parameters (weight and length) before the beginning of the experimental diet (3 mpf), after 2 months (5 mpf), and 4.5 months (7.5 mpf). (**B**) Growth parameters comparison divided by sex at 5 and 7.5 mpf. Black dot lines in the violin plots represent median (wider) and quartiles (thinner). (**C**) Growth parameters evolution (mean values ± s.e.m.) during the experiment for the three experimental groups. “CONTROL”, “MALTO”, and “PROBIO” refer to the experimental groups: control diet-fed, maltodextrin-fed, and probiotics-fed, respectively. Asterisks show statistically significant differences: * (*p* < 0.0500), ** (*p* < 0.0100).

In order to investigate the effects of probiotic supplementation in zebrafish on growth parameters, we weighted and measured all animals included in the experiment at t = 3 mpf (as an initial control at the beginning of the experiment), at t = 5 mpf (approximately the middle of the experiment), and at t = 7.5 mpf (ending of the experiment) (Figure 1). As expected, taking into account that the culture conditions were exactly the same and the animals came from the same batch, biometric analysis revealed no statistical differences (*p* > 0.0500) at 3 mpf. These data provided robust control values for the initial homogeneous groups (Figure 2A; t = 3 mpf). After two months of supplement ingestion, a significant increment was reported in terms of weight comparing probiotic-fed animals to control (*p* < 0.0500) and vehicle (*p* < 0.0500) groups (Figure 2A; t = 5 mpf). Similarly, length reported the same significant results (Figure 2A). The highest length values were reported in the PROBIO experimental group with 3.250 ± 0.0442 cm (mean ± s.e.m.), a result statistically different from CONTROL (*p* < 0.0100) and MALTO (*p* < 0.0100) groups. Interestingly, at 7.5 mpf, no significant differences (*p* < 0.0500) were reported among experimental groups on biometric analysis (Figure 2A; t = 7.5 mpf), suggesting that the probiotic-mixture treatment may induce a positive effect on younger stages of adult life that is overcome with time. 

When we analyzed the biometric results splitting data by sex, in the first evaluation after two months of experimental conditions, males included in the MALTO group showed statistical significant differences (*p* < 0.0500) compared to PROBIO in weight and length (Figure 2B; t = 5 mpf), reporting a clear effect of the anti-inflammatory and antioxidant probiotics on male growth. On the other hand, in females, data only reported statistical differences in standard length when PROBIO females were compared to both control groups (*p* < 0.0500) (Figure 2B; t = 5 mpf). Although statistical analysis did not report differences in female weight, it is interesting to notice that the female weight was over the mean values of controls in 90% of the studied animals within the PROBIO group. In the sex-split analysis at the ending of the experiment, only the PROBIO males reported a higher length (*p* < 0.0500) compared to maltodextrin-fed ones (Figure 2B; t = 7.5 mpf). 

The results show a strong positive effect of probiotic ingestion on growth parameters along the experimental time (Figure 2C) with a noticeable outcome in young individuals. Growth–length correlation was strong, as expected, reporting consistent Pearson r values (*r* > 0.7) (Supplementary File 1). It is interesting to notice that probiotic-fed animals showed the most homogenous correlation among samplings (Figure S1).

### 3.2. Effects of Probiotic Ingestion on Progenies

**Figure 3:**
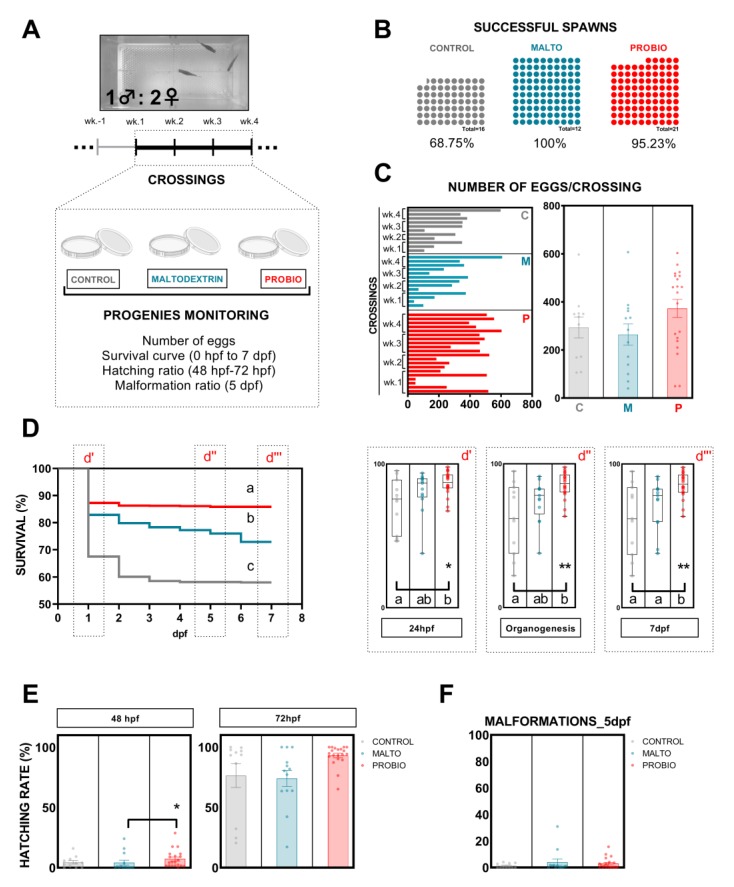
Probiotic ingestion positively affects zebrafish reproduction in terms of egg production and progeny quality. (**A**) Diagram summarizing design and all parameters analyzed involving each progeny. (**B**) The effect of experimental diets on the percentage of successful spawns, (**C**) total number of eggs spawned per crossing, (**D**) F1 survival (Kaplan–Meier curves (0–7dpf) and Box graphs (whiskers show min. to max.) at different key temporal points), (**E**) hatching rate at 48 hpf and 72 hpf, and (**F**) malformation rate. Data are presented as means ± s.e.m. “CONTROL/C”, “MALTO/M”, and “PROBIO/P” refer to the experimental groups: control diet-fed, maltodextrin-fed, and probiotics-fed, respectively. Asterisks show statistically significant differences: * (*p* < 0.0500), ** (*p* < 0.0100), and letters (a, b, c) show statistically significant differences among groups.

In our experiment, we performed five weekly crossings. Resulting progenies from crossings were monitored attending to different parameters (Figure 3A): number of successful spawns, number of eggs/crossing, survival curve, hatching rate, and malformation rate.

To discard the bias produced by first ever sexual intercourse (first gamete release), the first crossing was not evaluated or included within the experiment (Figure 3A). Control-fed animals showed the lowest percentage of successful spawns (68.75%) against the high percentages reported by both supplemented groups, maltodextrin (100%) and probiotic (95.23%) (Figure 3B). 

The number of eggs per crossing released by females are included in Figure 3C. Probiotic-fed animals reported earlier and, in general, higher numbers of eggs per crossing. The 80% (16/20) of the crossings included in this group registered more than 200 eggs, a higher percentage than the values shown by CONTROL and MALTO groups with 63.64% (8/13) and 61.54% (7/11), respectively (Figure 3C). However, no statistically significant differences were revealed when mean values were compared for each group (Figure 3C).

Significant differences (*p* < 0.0001) were revealed after the analysis of survival curves using the Kaplan–Meier method (Figure 3D). Progenies from each group showed a different survival figure. Notably, the comparison of mean values showed a statistical difference between F1 derived from control-fed and probiotic-fed animals from 24 hpf to the end of evaluation at 7 dpf (Figure 3D; d’, d’’, and d’’’). The vehicle control, MALTO, only revealed statistical differences with control at the last day of evaluation (Figure 3D-d’’’). At 7 dpf, the final survival was in the PROBIO group with 85.19 ± 2.037%, which was 23.54% and 13.35% higher than the F1 CONTROL and MALTO progenies, respectively.

The usual survival drop at 24 hpf was strongly attenuated by the ingestion of the probiotic strains and the number of viable larvae remained almost constant from this point to the end of the survival evaluation between progenies derived from controls progenitors and those from probiotic-fed ones (Figure 3D). These results indicate a conclusive effect of parental probiotic ingestion on progeny development. Moreover, hatching rate reported a positive nonstatistical tendency in F1-PROBIO larvae (Figure 3E), whereas the number of malformed larvae did not vary among groups at 5 dpf (Figure 3F), supporting the use of the probiotic supplement. 

### 3.3. Effects of Probiotic Ingestion on Concentration, Total Sperm Motility, and Progressive Motility

Sperm was collected from all males included in this study at two key times: 5 and 7.5 mpf concurring with 15 days before and after crossings were performed. Results regarding number of spermiating males, sperm concentration, total motility, progressive motility, and sperm kinetics for these two samplings are presented in Figure 3. 

**Figure 4:**
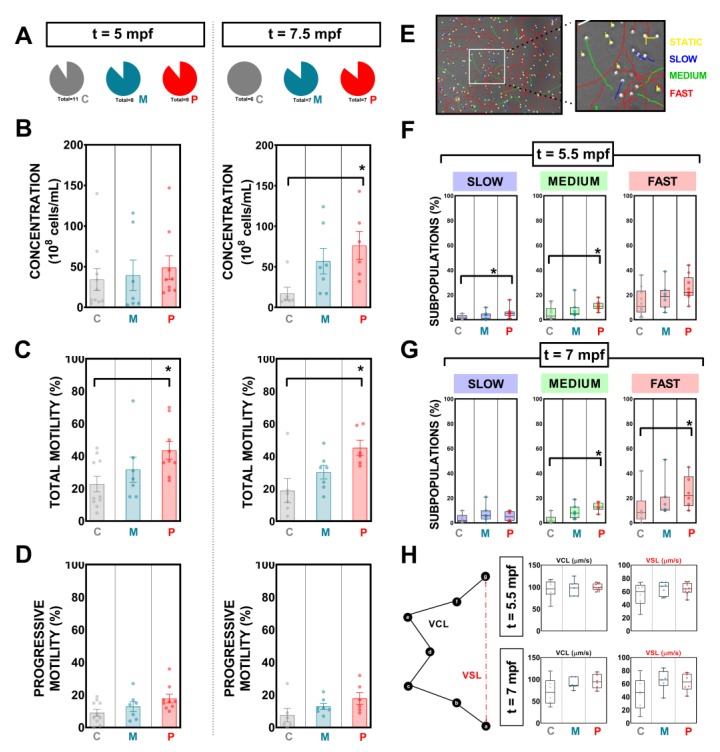
Mixture supplementation effects on zebrafish sperm kinematics after a long exposure of ingestion. (**A**) Percentage of spermiating males at the two studied times. (**B**) Concentration, (**C**) total motility, and (**D**) progressive motility at 5 and 7.5 mpf reported for each experimental group. (**E**) Example of a CASA field rendering static, slow, medium, and fast subpopulations. (**F**) Sperm subpopulations within the motile population according to speed parameters at 5 and 7.5 mpf (**G**) for each group. (**H**) Diagram describing curvilinear and straight velocities and box graphs (whiskers show min. to max.) at different key temporal points showing results from each experimental group. Data are presented as means ± s.e.m. “C”, “M”, and “P” refer to the experimental groups: control diet-fed, maltodextrin-fed, and probiotics-fed, respectively. Asterisks show statistically significant differences: * (*p* < 0.0500).

Overall, the number of fluent males producing sperm was similar among groups at both evaluation times (Figure 4A). A period of two months of probiotic ingestion did not modify (*p* < 0.0050) sperm count (10^8^ cells/mL; mean ± s.e.m.) in the PROBIO group (Figure 4B; 5 mpf) when compared to both control groups. After crossings, in the second evaluation at 7.5 mpf, the mean value for total sperm counts in the PROBIO group was 76.17 ± 17.18, a result statistically different from CONTROL males, which reported the lowest mean values for concentration: 17.00 ± 7.84 (Figure 4B; 7.5 mpf). 

Concerning total motility (%; mean ± s.e.m.), controls (standard diet and vehicle) showed similar values (*p* > 0.0500) at 5 mpf sampling. However, males from the PROBIO group registered higher percentages of motile cells (43.56 ± 5.419) when compared to CONTROL ones (22.80 ± 4.825) (Figure 4C; 5 mpf). A similar scenario was shown at 7.5 mpf with 45.17 ± 4.679 and 18.83 ± 7.427 for males fed with probiotic strains and standard diet, respectively (Figure 4C; 7.5 mpf).

In contrast, the animals with a feeding regime supplemented with probiotics did not reveal any statistical difference (*p* < 0.0500) on progressive motility at any of both samplings, reporting similar mean values (Figure 4D).

In the present study, four fractions of motile cells were created and set up in CASA software in terms of curvilinear velocity (VCL): static, slow, medium, and fast, according to the following thresholds: 10 µm/s < slow < 45 µm/s < medium < 100 µm/s < fast (Figure 4E). Interestingly, PROBIO males showed statistical differences after two months of strains ingestion in slow and medium subpopulations when compared to control-fed animals (Figure 4F). At 7.5 mpf, probiotic-fed males reported higher percentages of fast and medium cells when compared to control samples (Figure 4G).

We inspected sperm kinematic parameters, where there was no overall difference (*p* > 0.0500) among experimental groups. Figure 4H shows the results for sperm curvilinear velocity (VCL) and straight velocity (VSL). Principal component analysis (PCA) results (CONTROL vs PROBIO) from CASA variables can be found in Figure S2.

## 4. Discussion

In this study, we carried out an evaluation of a long exposure to a diet supplemented with probiotics on sperm quality and progeny development. For this purpose, we have used zebrafish (*Danio rerio*) as a teleost model. This small vertebrate has been described as a versatile animal model for fertility research [16]. In light of the early evidence about the use of probiotics for infertility treatment [26,27,28] and the publication of a theory linking gut health with testicular function [29,30], we hypothesize that probiotic strains may have an effect on reproductive performance after a long exposure of ingestion in the animal production field. The microbiome (the genes and genomes of the microbiota, as well as the products of the microbiota and the host environment [31]) is crucial in several essential functions within the organism, including supporting digestion and metabolism, keeping the gut barrier integrity, producing vitamins, and maintaining the immune system [32,33]. In the periconceptional period, male and female reproductive microbes play a key role in fertility and they can have an impact during conception [34]. Probiotics are starting to emerge as new tools for overcoming reproductive failure [34].

Supporting our hypothesis are some previous results from our group on the beneficial use of probiotics in the zebrafish model [26,28]. In the present study, two probiotic strains—*Lactobacillus rhamnosus* CECT8361 and *Bifidobacterium longum* CECT7347—were vehicled in maltodextrin and provided to young animals for a total period of 3.5 months (Figure 1). The selected *Bifidobacterium* strain has been previously described as a bacteria with anti-inflammatory activity [23,35]. Furthermore, the combination of both used strains has reported a synergic antioxidant activity [22]. In addition, *Lactobacillus* and *Bifidobacterium* are both included within the most used genera as probiotics at the present time [36]. This evidence, together with the simplicity and acceptability of the probiotics format, makes the use of these strains a potential interesting supplement in the reproductive biotechnology field and therefore we considered it interesting to evaluate the effects of these beneficial microbes. 

The diet of teleost broodstock is extremely important, not merely to its health state but also to gametes (the spermatozoa being the most sensitive cell to imbalances due to their long maturation progress) and progeny quality [37]. As a first evidence of positive effect of probiotic ingestion, our model animal reported a growth response (Figure 2). Length and weight were significantly promoted by bacteria feeding in both sexes, finding a superior growth performance in males (Figure 2). Probiotics, as nutritional supplementation, have been shown to exert and impact growth in zebrafish [38]. The resulting increases in growth rate in the fish receiving probiotics may be led by a more efficient use of diet-derived energy sources.

With the objective of evaluating couple-based outcomes, we crossed exposed animals and studied the offspring (Figure 3). This aspect represents a far more critical output since reproductive function ideally should be consistent and therefore predictable over the long term. Fecundity increased in our experiment in those animals fed with probiotics, reporting higher numbers of eggs per crossing since the first attempts of intercourse when compared with control groups (Figure 3). A stimulatory role of a probiotic lactic bacteria (*Lactobacillus rhamnosus)* on female zebrafish fecundity has been previously reported [39]. Authors suggest that the positive effect of this lactic bacteria is due to the activation of the endocrine control and to the direct action of leptin on the ovary after performing gene expression analysis of neuropeptide hormones and metabolic signals, such as *kiss1*, *kiss2*, and *leptin* both at the CNS level and at the peripheral level [39]. Moreover, the best F1 survival percentages at 7 dpf were registered in larvae derived from PROBIO-fed progenitors (Figure 3C-d’’’). The most notable finding of this study regards male gamete quality promotion (Figure 4). In the present study, the sperm concentration of fluent male zebrafish fed with the probiotic-supplemented diet was significantly higher than in control groups after a long exposure (3.5 months) (Figure 4). Sperm count is closely linked to male fecundity and is an essential parameter of semen analysis, the first step to identify male factor infertility in humans [40,41] or teleost fish [42]. These results are in agreement with those of a previous study in humans in which oligo asthenospermic men improved sperm counts after a prolonged ingestion (one year) of a commercial symbiotic (formulations combining probiotics and prebiotics) [27]. Sperm quality was also improved in terms of motility after the ingestion of *L. rhamnosus* CECT8361 and *B. longum* CECT7347. We registered a total motility improvement in PROBIO fluent males already after two months of supplemented-diet ingestion (Figure 4) which was maintained over time until the second sampling after crossings after 3.5 months of probiotic feeding. This supports the observations previously demonstrated by our lab for short ingestion of a higher dose per animal of this probiotic mixture supplement in the zebrafish model [28], suggesting that the consistent application of these probiotic strains, as part of the routine feeding diet regime, also enhances male gamete quality. We speculate that the enhanced availability of nutrients derived by bacteria ingestion facilitated a more efficient nutrient absorption by the gastrointestinal tract. This must be considered together with the proven antioxidant and anti-inflammatory activities of the studied strains.

## 5. Conclusions

The findings included in the present trial demonstrate the beneficial effects of prolonged oral ingestion of a mixture (1:1) of *Lactobacillus rhamnosus* CECT8361 and *Bifidobacterium longum* CECT7347 supplementation on zebrafish reproductive performance and confirm previous data from our laboratory [22,26]. A small dose per individual of the probiotic mixture proved beneficial effects in terms of growth parameters, better sperm quality, spawning, and progeny survival. Concretely, the probiotic strains seem to have an impact on sperm count and total sperm motility. The strain ingestion promoted fast sperm subpopulations and number of released eggs per crossings as well as a better F1 larvae survival. From the animal production point of view, the findings we report with the zebrafish model provide novel insights into the role exerted by probiotics on vertebrate reproductive performance favoring the exploration of new successful technologies for the animal production industry.

## Supplementary Materials

The following are available online at https://www.mdpi.com/2218-273X/9/8/338/s1, Figure S1: Growth parameters (length–weight) correlations and statistics for each group at 5 and 7.5 mpf. “CONTROL”, “MALTO”, and “PROBIO” refer to the experimental groups: control diet-fed, maltodextrin-fed, and probiotics-fed, respectively. Figure S2: PCA analysis for computer-assisted sperm analysis (CASA) variables for control and probiotic-fed group. Representation of the experimental group in a principal component plane at (A) 5 mpf and (B) 7.5 mpf.

## References

[1] Howe K., Clark M.D., Torroja C.F., Torrance J., Berthelot C., Muffato M., Collins J.E., Humphray S., McLaren K., Matthews L. (2013). The zebrafish reference genome sequence and its relationship to the human genome. Nature.

[2] Broughton R.E., Milam J.E., Roe B.A. (2001). The Complete Sequence of the Zebrafish (*Danio rerio*) Mitochondrial Genome and Evolutionary Patterns in Vertebrate Mitochondrial DNA. Genome Res..

[3] Golling G., Amsterdam A., Sun Z., Antonelli M., Maldonado E., Chen W., Burgess S., Haldi M., Artzt K., Farrington S. (2002). Insertional mutagenesis in zebrafish rapidly identifies genes essential for early vertebrate development. Nat. Genet..

[4] Grunwald D.J., Eisen J.S. (2002). Headwaters of the zebrafish -- emergence of a new model vertebrate. Nat. Rev. Genet..

[5] Stewart A.M., Braubach O., Spitsbergen J., Gerlai R., Kalueff A.V. (2014). Zebrafish models for translational neuroscience research: from tank to bedside. Trends Neurosci..

[6] MacRae C.A., Peterson R.T. (2015). Zebrafish as tools for drug discovery. Nat. Rev. Drug Discov..

[7] Bambino K., Chu J. (2017). Zebrafish in Toxicology and Environmental Health. Curr. Top. Dev. Biol..

[8] Keller E.T., Murtha J.M. (2004). The use of mature zebrafish (*Danio rerio*) as a model for human aging and disease. Comp. Biochem. Physiol. C. Toxicol. Pharmacol..

[9] Lieschke G.J., Currie P.D. (2007). Animal models of human disease: zebrafish swim into view. Nat. Rev. Genet..

[10] Riesco M.F., Valcarce D.G., Alfonso J., Herráez M.P., Robles V. (2014). In vitro generation of zebrafish PGC-like cells. Biol. Reprod..

[11] Lee O., Takesono A., Tada M., Tyler C.R., Kudoh T. (2012). Biosensor zebrafish provide new insights into potential health effects of environmental estrogens. Environ. Health Perspect..

[12] Zhang Z., Lau S.-W., Zhang L., Ge W. (2015). Disruption of Zebrafish Follicle-Stimulating Hormone Receptor (*fshr*) But Not Luteinizing Hormone Receptor (*lhcgr*) Gene by TALEN Leads to Failed Follicle Activation in Females Followed by Sexual Reversal to Males. Endocrinology.

[13] Darrow K.O., Harris W.A. (2004). Characterization and development of courtship in zebrafish, Danio rerio. Zebrafish.

[14] Laan M., Richmond H., He C., Campbell R.K. (2002). Zebrafish as a Model for Vertebrate Reproduction: Characterization of the First Functional Zebrafish (*Danio rerio*) Gonadotropin Receptor. Gen. Comp. Endocrinol..

[15] Blanton M.L., Specker J.L. (2007). The hypothalamic-pituitary-thyroid (HPT) axis in fish and its role in fish development and reproduction. Crit. Rev. Toxicol..

[16] Hoo J.Y., Kumari Y., Shaikh M.F., Hue S.M., Goh B.H. (2016). Zebrafish: A Versatile Animal Model for Fertility Research. Biomed Res. Int..

[17] Schagdarsurengin U., Steger K. (2016). Epigenetics in male reproduction: effect of paternal diet on sperm quality and offspring health. Nat. Rev. Urol..

[18] Salas-Huetos A., Bulló M., Salas-Salvadó J. (2017). Dietary patterns, foods and nutrients in male fertility parameters and fecundability: a systematic review of observational studies. Hum. Reprod. Update.

[19] Gaskins A.J., Colaci D.S., Mendiola J., Swan S.H., Chavarro J.E. (2012). Dietary patterns and semen quality in young men. Hum. Reprod..

[20] Nassan F.L., Chavarro J.E., Tanrikut C. (2018). Diet and men’s fertility: does diet affect sperm quality?. Fertil. Steril..

[21] Hill C., Guarner F., Reid G., Gibson G.R., Merenstein D.J., Pot B., Morelli L., Canani R.B., Flint H.J., Salminen S. (2014). The International Scientific Association for Probiotics and Prebiotics consensus statement on the scope and appropriate use of the term probiotic. Nat. Rev. Gastroenterol. Hepatol..

[22] Valcarce D.G., Genovés S., Riesco M.F., Martorell P., Herráez M.P., Ramón D., Robles V. (2017). Probiotic administration improves sperm quality in asthenozoospermic human donors. Benef. Microbes.

[23] Medina M., De Palma G., Ribes-Koninckx C., Calabuig M., Sanz Y. (2008). Bifidobacterium strains suppress in vitro the pro-inflammatory milieu triggered by the large intestinal microbiota of coeliac patients. J. Inflamm. (Lond)..

[24] Westerfield M. (2000). The Zebrafish Book. A Guide for the Laboratory Use of Zebrafish (Danio rerio).

[25] Kimmel C.B., Ballard W.W., Kimmel S.R., Ullmann B., Schilling T.F. (1995). Stages of Embryonic Development of the Zebrafish. Dev. Dyn..

[26] Valcarce D.G., Pardo M.Á., Riesco M.F., Cruz Z., Robles V. (2015). Effect of diet supplementation with a commercial probiotic containing *Pediococcus acidilactici* (Lindner, 1887) on the expression of five quality markers in zebrafish (*Danio rerio* (Hamilton, 1822)) testis. J. Appl. Ichthyol..

[27] Maretti C., Cavallini G. (2017). The association of a probiotic with a prebiotic (Flortec, Bracco) to improve the quality/quantity of spermatozoa in infertile patients with idiopathic oligoasthenoteratospermia: a pilot study. Andrology.

[28] Valcarce D.G., Riesco M.F., Martínez-Vázquez J.M., Robles V. (2019). Diet Supplemented with Antioxidant and Anti-Inflammatory Probiotics Improves Sperm Quality after Only One Spermatogenic Cycle in Zebrafish Model. Nutrients.

[29] Tremellen K. (2016). Gut Endotoxin Leading to a Decline IN Gonadal function (GELDING) - a novel theory for the development of late onset hypogonadism in obese men. Basic Clin. Androl..

[30] Tremellen K., Pearce K. (2017). Probiotics to improve testicular function (Andrology 5:439-444, 2017) - a comment on mechanism of action and therapeutic potential of probiotics beyond reproduction. Andrology.

[31] Whiteside S.A., Razvi H., Dave S., Reid G., Burton J.P. (2015). The microbiome of the urinary tract—a role beyond infection. Nat. Rev. Urol..

[32] Rautava S., Luoto R., Salminen S., Isolauri E. (2012). Microbial contact during pregnancy, intestinal colonization and human disease. Nat. Rev. Gastroenterol. Hepatol..

[33] Gensollen T., Iyer S.S., Kasper D.L., Blumberg R.S. (2016). How colonization by microbiota in early life shapes the immune system. Science (80-. ).

[34] Selma-Royo M., Tarrazó M., García-Mantrana I., Gómez-Gallego C., Salminen S., Collado M.C. (2019). Shaping Microbiota During the First 1000 Days of Life. Adv. Exp. Med. Biol..

[35] De Palma G., Kamanova J., Cinova J., Olivares M., Drasarova H., Tuckova L., Sanz Y. (2012). Modulation of phenotypic and functional maturation of dendritic cells by intestinal bacteria and gliadin: relevance for celiac disease. J. Leukoc. Biol..

[36] Fijan S. (2014). Microorganisms with claimed probiotic properties: an overview of recent literature. Int. J. Environ. Res. Public Health.

[37] Sinclair K.D., Watkins A.J. (2014). Parental diet, pregnancy outcomes and offspring health: metabolic determinants in developing oocytes and embryos. Reprod. Fertil. Dev..

[38] Carnevali O., Avella M.A., Gioacchini G. (2013). Effects of probiotic administration on zebrafish development and reproduction. Gen. Comp. Endocrinol..

[39] Gioacchini G., Maradonna F., Lombardo F., Bizzaro D., Olivotto I., Carnevali O. (2010). Increase of fecundity by probiotic administration in zebrafish (Danio rerio). Reproduction.

[40] World Health Organization (2010). WHO Laboratory Manual for the Examination and Processing of Human Semen.

[41] Wang C., Swerdloff R.S. (2014). Limitations of semen analysis as a test of male fertility and anticipated needs from newer tests. Fertil. Steril..

[42] Kowalski R.K., Cejko B.I. (2019). Sperm quality in fish: Determinants and affecting factors. Theriogenology.

