# Supplementary material for: Long Exposure to a Diet Supplemented with Antioxidant and Anti-Inflammatory Probiotics Improves Sperm Quality and Progeny Survival in the Zebrafish Model
Source: Biomolecules. 2019 Aug 3;9(8):338. doi: 10.3390/biom9080338 (PMC6724062; doi:10.3390/biom9080338)
Supplement: Supplementary file 1 [file biomolecules-09-00338-s001.pdf]

Supplementary Materials

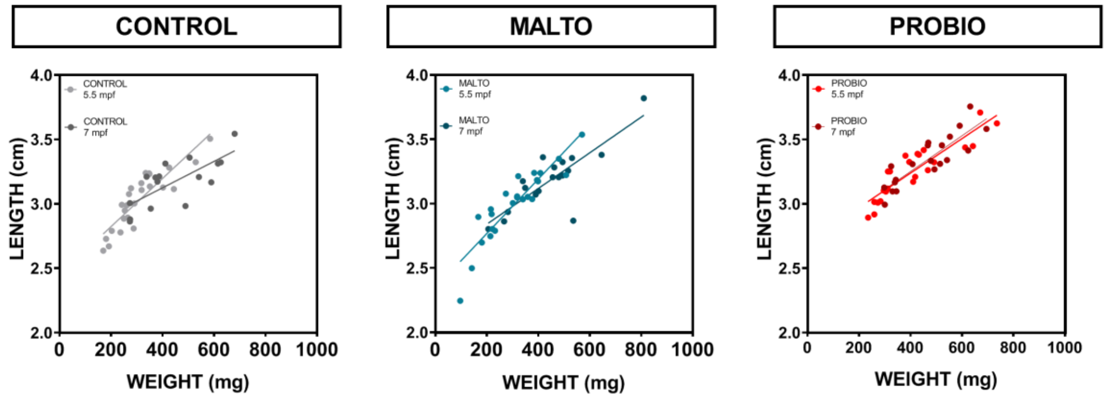

5 mpf

CONTROL

Pearson r	
r	0.8767
95% confidence interval	0.7368 to 0.9446
R squared	0.7686
P value	
P (two-tailed)	<0.0001
P value summary	****

MALTO

Pearson r	
r	0.9059
95% confidence interval	0.7919 to 0.9589
R squared	0.8207
P value	
P (two-tailed)	<0.0001
P value summary	****

PROBIO

Pearson r	
r	0.8964
95% confidence interval	0.7679 to 0.9555
R squared	0.8035
P value	
P (two-tailed)	<0.0001
P value summary	****

7.5 mpf

CONTROL

Pearson r	
r	0.7381
95% confidence interval	0.3823 to 0.9033
R squared	0.5449
P value	
P (two-tailed)	0.0011
P value summary	**

MALTO

Pearson r	
r	0.8223
95% confidence interval	0.5770 to 0.9316
R squared	0.6762
P value	
P (two-tailed)	<0.0001
P value summary	****

PROBIO

Pearson r	
r	0.8645
95% confidence interval	0.6834 to 0.9454
R squared	0.7473
P value	
P (two-tailed)	<0.0001
P value summary	****

Figure S1. Growth parameters (length–weight) correlations and statistics for each group at 5 and 7.5 mpf. “CONTROL”, “MALTO”, and “PROBIO” refer to the experimental groups: control diet-fed, maltodextrin-fed, and probiotics-fed, respectively.

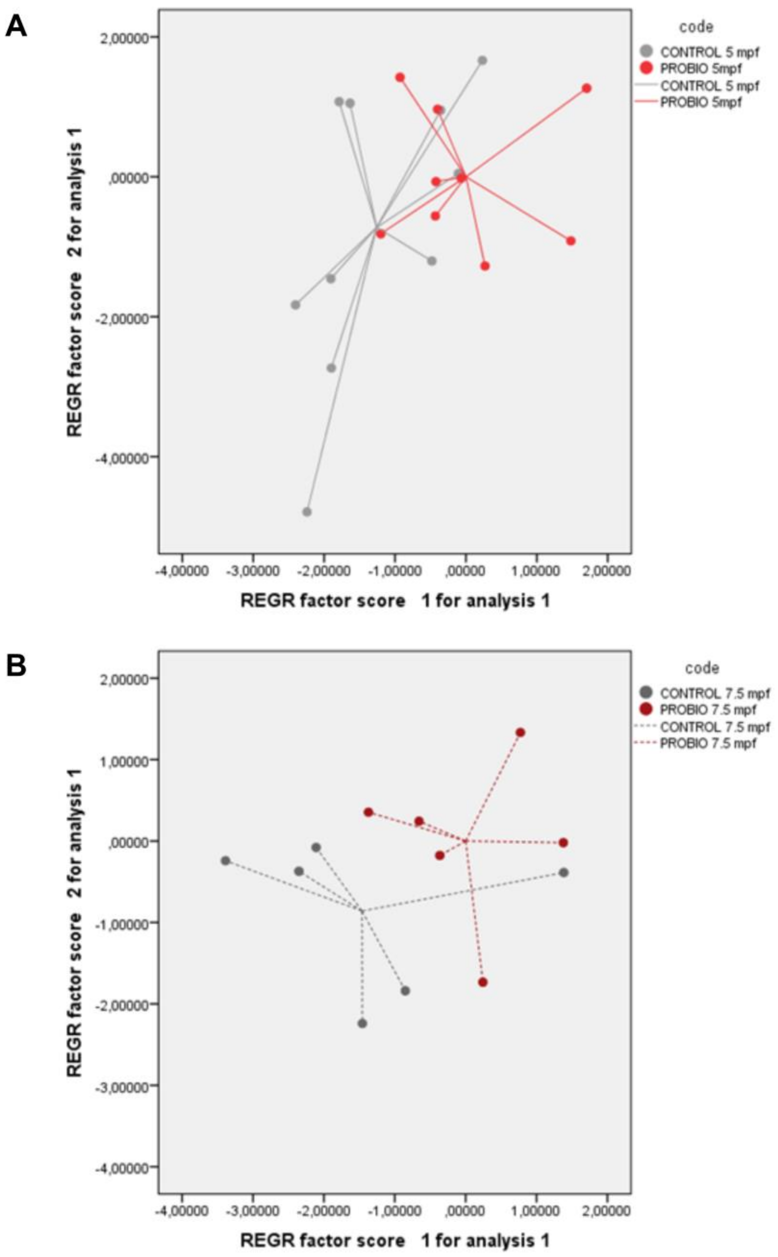

Figure S2. PCA analysis for computer-assisted sperm analysis (CASA) variables for control and probiotic-fed group. Representation of the experimental group in a principal component plane at (**A**) 5 mpf and (**B**) 7.5 mpf.
